# Activation of NLRP3 inflammasome in stable chronic obstructive pulmonary disease

**DOI:** 10.1038/s41598-022-11164-1

**Published:** 2022-05-09

**Authors:** Ivona Markelić, Iva Hlapčić, Andrea Čeri, Margareta Radić Antolic, Miroslav Samaržija, Sanja Popović-Grle, Andrea Vukić Dugac, Lada Rumora

**Affiliations:** 1 Clinic for Respiratory Diseases Jordanovac, University Hospital Centre Zagreb, Zagreb, Croatia; 2 Department of Medical Biochemistry and Haematology, Faculty of Pharmacy and Biochemistry, University of Zagreb, Zagreb, Croatia; 3 Clinical Institute of Laboratory Diagnostics, University Hospital Centre Zagreb, Zagreb, Croatia; 4 School of Medicine, University of Zagreb, Zagreb, Croatia

**Keywords:** Cytokines, RNA, Biochemistry, Biomarkers, Diseases, Respiratory tract diseases, Chronic obstructive pulmonary disease

## Abstract

Nucleotide-binding oligomerization domain-like receptor family pyrin domain-containing 3 (NLRP3) inflammasome activation plays an important role in chronic obstructive pulmonary disease (COPD) pathogenesis and might be involved in ongoing chronic inflammation. This study aimed to determine interleukin-1beta (IL-1β) plasma concentration as well as *IL1B*, *NLRP3* and caspase-1 (*CASP1*) gene expression in the Croatian COPD patients. 109 patients with stable COPD and age- and sex-matched 95 controls were included in the study. Plasma IL-1β concentration was measured by Luminex technology, and gene expression analysis was performed using TaqMan assays. It was shown that COPD patients had increased concentration of IL-1β and enhanced gene expression of *IL1B*, *NLRP3* and *CASP1* compared to controls. There was no difference in IL-1β or *IL1B*, *NLRP3* and *CASP1* in patients with COPD regarding airflow obstruction severity and smoking history. Finally, the diagnostic potential of the determined parameters was evaluated, and it was found that IL-1β correctly classified 89% of cases in the combination with common inflammatory biomarkers, white blood cell count and fibrinogen, showing a potential in COPD prediction. In conclusion, up-regulation of *IL1B*, *NLRP3*, *CASP1* and increased IL-1β concentration suggest the activation of NLRP3 inflammasome in the systemic compartment of patients with stable COPD.

## Introduction

According to the latest data from the World Health Organization, chronic obstructive pulmonary disease (COPD) is the third leading cause of death worldwide^1^, with a projected increase in social, economic and health care burden^2^. COPD is a progressive lung disease characterized by persistent respiratory symptoms and airflow limitation due to the airway and/or alveolar abnormalities that are usually caused by a significant exposure to noxious particles or gases^3^. The immune response in COPD is altered, and chronic inflammation is known to be associated with the development of COPD. Regardless numerous studies, further investigation is recommended to elucidate the inflammatory response in COPD.

Activation of inflammasomes is considered to be involved in the pathogenesis of COPD. Among the various inflammasomes, which are activated by different agonists, the most studied one is the nucleotide-binding oligomerization domain-like receptor family pyrin domain-containing 3 (NLRP3) inflammasome. The NLRP3 inflammasome is a multimeric complex involved in the release of the pro-inflammatory cytokines interleukin (IL)-1β and IL-18. It consists of the sensor NLRP3 protein, the adaptor named apoptosis-associated speck-like protein containing a caspase activation and recruitment domain (ASC) and the effector, which is in most cases pro-caspase-1^4^. There are different pathways for NLRP3 inflammasome activation described in the literature. Those are classical (canonical and non-canonical) as well as alternative pathways. NLRP3 inflammasome activation is usually considered to be a two-step process, consisting of priming and activation (classical pathways). The first step involves sensing and producing, which begins with the recognition of pathogen-associated molecular patterns and/or damage-associated molecular patterns by pattern recognition receptors, such as Toll-like receptors (TLRs)^5^. Stimulation of TLRs leads to activation of nuclear factor kappa B (NF-κB)-mediated signalling. Activated NF-κB signalling pathway results in increased transcription and production of NLRP3 protein, pro-IL-1β and pro-IL-18, which finally leads to the priming or initiation of the NLRP3 inflammasome activation^6^. The second step begins with the assembly of the NLRP3 inflammasome, where ASC acts as a zipper and binds NLRP3 with pro-caspase-1, which in turn undergoes proteolytic cleavage that releases caspase-1. On the other hand, caspases-4/5 in humans and caspase-11 in mice are involved in non-canonical pathway that is triggered by bacterial infection i.e. by the internalization of lipopolysaccharide into the cytosol. Active caspase-1 (or caspase-4/5/11) is able to process pro-IL-1β and pro-IL-18 into their mature forms that induce inflammation and pyroptotic cell death^4–7^. In contrast, only one signal provokes NLRP3 inflammasome activation in the alternative pathway that ultimately leads to inflammation without pyroptosis^8^.

Until now, investigation of the role of NLRP3 inflammasome in COPD has been explored in animal models and human respiratory system samples^9–11^, but there is limited information on the NLRP3 inflammasome activation in the peripheral circulation of COPD patients. Besides, activation of the NLRP3 inflammasome is found to be involved in the immune response to cigarette smoke, which is the major environmental risk factor for the onset of COPD^12^.

The chronic systemic inflammation in COPD and the altered immune response regulated by the activation of NLRP3 inflammasome might play an important role in COPD pathogenesis. Therefore, this study investigated the plasma concentration of IL-1β as well as IL-1β gene (*IL1B)*, *NLRP3* and caspase-1 gene (*CASP1*) expression in the Croatian COPD patients in stable phase in comparison to the healthy controls. The study also aimed to determine the association between the aforementioned parameters and airflow obstruction severity or smoking history.

## Materials and methods

### Participants

The current retrospective case–control study included 109 COPD patients in a stable phase of the disease and 95 age- and sex-matched healthy individuals who voluntarily participated in the study and signed an informed consent form. The recruitment of the participants was conducted at Clinic for Respiratory Diseases Jordanovac, University Hospital Centre Zagreb in 2017 and 2018. The study was approved by the Ethics Committee of University Hospital Centre Zagreb (Zagreb, Croatia) and by the Ethics Committee for Experimentation of the University of Zagreb Faculty of Pharmacy and Biochemistry (Zagreb, Croatia) (approval protocol numbers: 02/21/JG and 251-62-03-14-78, respectively). COPD diagnosis was confirmed by pulmonology specialists according to the guidelines of Global Initiative for Chronic Obstructive Pulmonary Disease (GOLD)^3^, after measuring the spirometry parameters forced expiratory volume in the first second (FEV_1_) and forced vital capacity (FVC). Healthy individuals were included in the study based on medical history data and spirometry results. Based on FEV_1_, there were 39 COPD patients in GOLD 2, 36 COPD patients in GOLD 3 and 34 COPD patients in GOLD 4 group. Self-reported smoking history data was collected so that there were 48 healthy non-smokers, 47 healthy smokers, 5 COPD non-smokers, 75 COPD former smokers and 29 COPD smokers. Both COPD patients and healthy subjects were older than 40 years, had no lung disease (except COPD in COPD patients), inflammatory systemic diseases, acute infections, diabetes with severe complications, severe liver disease, severe renal insufficiency, malignant diseases, transplantation, and other specific or non-specific acute inflammation.

### Common inflammatory biomarkers

Blood samples were collected by venepuncture of a large antecubital vein after overnight fasting from 7 a.m. to 9 a.m. Tubes containing K_3_-ethylenediaminetetraacetic acid (K_3_ EDTA) as an anticoagulant (Greiner Bio-One, Kremsmünster, Austria) were used for white blood cells (WBC) count and determination of IL-1β, and for extraction of buffy coat, which was used for RNA and DNA isolation. A tube containing 3.2% sodium citrate (Becton, Dickinson and Company, Franklin Lakes, NJ, USA) was used for fibrinogen (FIB) measurement, while a tube without additive but with clot activator and gel separator was used for C-reactive protein (CRP) determination (Greiner Bio-One, Kremsmünster, Austria)^13^. Each sample was further treated according to the recommendations^14^. Determination of WBC, FIB and CRP was performed at Clinical Institute of Laboratory Diagnostics at University Hospital Centre Zagreb (Zagreb, Croatia), as described previously^15^.

### IL-1β determination

EDTA plasma was used for determination of IL-1β concentration using Procarta Plex High Sensitivity Luminex kit (Thermo Fischer Scientific, Waltham, MA, USA), according to the manufacturer’s recommendations. The results were analysed using a Luminex 200 instrument, and the IL-1β concentration was assessed from a standard curve using the xPONENT software package (Luminex Corporation, Austin, TX, USA).

### Gene expression analysis

Total RNA was isolated from buffy coat by the TRIzol/chloroform method^16^, and the quality of the RNA was assessed by the ratio of the 260/280 nm measurements on microvolume spectrophotometer Nanodrop 8000 (Thermo Fischer Scientific, Wilmington, USA). When the ratio was 1.9–2.1, the RNA was suitable for cDNA synthesis by reverse transcription based on polymerase chain reaction using RevertAid First Strand cDNA Synthesis Kit (Thermo Fischer Scientific, Waltham, Massachusetts, USA) with GeneAmp PCR System 9700 (Applied Biosystems, Foster City, USA)^17^. The assessment of *IL1B*, *NLRP3* and *CASP1* gene expressions were performed by TaqMan Gene Expression Assays (Hs01555410_m1 for *IL1B*, Hs00918082_m1 for *NLRP3*, Hs00354836_m1 for *CASP1*; Applied Biosystems, Foster City, USA) and TaqMan Universal Master Mix (Applied Biosystems, Foster City, USA) according to the manufacturer’s guidelines. Data were normalized using gene expression of beta-2-microglobulin (*B2M*) and peptidylprolyl isomerase (*PPIA*) (Hs99999907_m1 for *B2M*, Hs99999904_m1for *PPIA*; Applied Biosystems, Foster City, USA) as the endogenous controls. In addition, a randomly selected control sample was included in each plate as a calibrator. Finally, the relative expression of target genes in controls and COPD patients was performed using the 2^−∆∆Ct^ comparison method^18^.

### Statistics

All data failed a normality test performed by Kolmogorov–Smirnov test, so the results were shown as a median with corresponding interquartile range (IQR), beside age that was shown as a median with minimum and maximum and sex that was shown in absolute numbers. Non-parametric Mann–Whitney test and Kruskal–Wallis test were used to test the differences between the groups of interest, while categorical data was tested by Chi-squared test. Univariate and multivariable logistic regression analyses were performed to assess the predictor variables on the risk of COPD, so results were shown as odds ratio (OR) with corresponding 95% confidence interval (CI). Influence of age, sex, comorbidities and therapy was excluded in this investigation. Analysis was performed in MedCalc statistical software version 17.9.2. (MedCalc Software, Ostend, Belgium). Results were statistically significant if *P* < 0.05.

### Informed consent statement

Informed consent was obtained from all subjects involved in the study.

## Results

### Characteristics of the study participants

109 COPD patients and 95 healthy volunteers were included in the present study. A brief overview of all participants including basic characteristics, spirometry and common inflammatory parameters are shown in Table 1. There are no differences between controls and COPD patients regarding age and sex distribution, while lung function parameters were decreased in COPD patients (*P* < 0.001). Markers of systemic inflammation, CRP, FIB and WBC were significantly increased in COPD patients.

**Table 1:** Basic characteristics, spirometry and common inflammatory markers of all participants included in the study.

Parameter	Controls (n = 95)	COPD patients (n = 109)	*P* value
Age	64 (46–83)	65 (45–87)	0.069
**Sex**			
Male	49	69	0.121
Female	46	40	
FEV_1_ (L)	2.60 (2.12–3.19)	1.08 (0.69–1.60)	**< 0.001**
FEV_1_ (% pred.)	93.3 (86.4–104.2)	40.8 (27.9–61.7)	**< 0.001**
FVC (L)	3.35 (2.77–4.16)	2.28 (1.74–2.77)	**< 0.001**
FEV_1_/FVC (%)	80.6 (76.8–87.6)	51.3 (40.7–58.7)	**< 0.001**
CRP (mg/L)	1.47 (0.74–2.78)	2.34 (1.15–5.67)	**< 0.001**
FIB (g/L)	3.5 (3.1–3.8)	3.8 (3.4–4.5)	**< 0.001**
WBC (× 10^9^/L)	6.14 (5.14–7.42)	7.57 (6.55–8.95)	**< 0.001**
**Smoking status**			
Current smokers	47	37	**< 0.001**
Former smokers	0	90	
Never smokers	48	10	

Age is presented as a median with minimum and maximum, and sex is presented as an absolute number.

Other parameters were shown as a median with IQR.

Data was analysed by Chi-squared or Mann–Whitney test, and results were considered statistically significant when *P* < 0.05.

Significant values are in bold.

*FEV*_*1*_ forced expiratory volume in the first second, *FVC* forced vital capacity, *CRP* C-reactive protein, *FIB* fibrinogen, *WBC* white blood cells, *COPD* chronic obstructive pulmonary disease.

### IL-1β concentration and *IL1B*, *NLRP3* and *CASP1* expression in the systemic compartment

**Figure 1:**
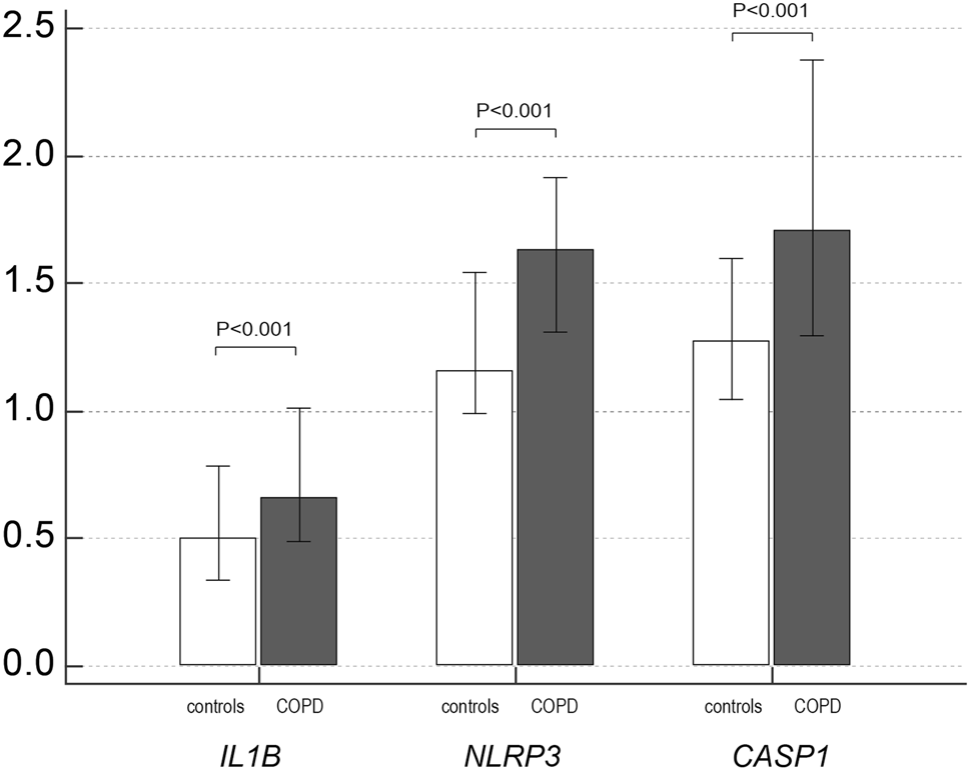
Relative gene expression of *IL1B*, *NLRP3* and *CASP1* in control and COPD groups. *IL1B* interleukin-1beta gene, *NLRP3* nucleotide-binding oligomerization domain-like receptor family pyrin domain-containing 3 gene, *CASP1* caspase-1 gene, *COPD* chronic obstructive pulmonary disease. Mann–Whitney test was used for comparison of *IL1B*, *NLRP3* and *CASP1* relative expression between controls and COPD patients. Results were presented as a median and IQR.

**Figure 2:**
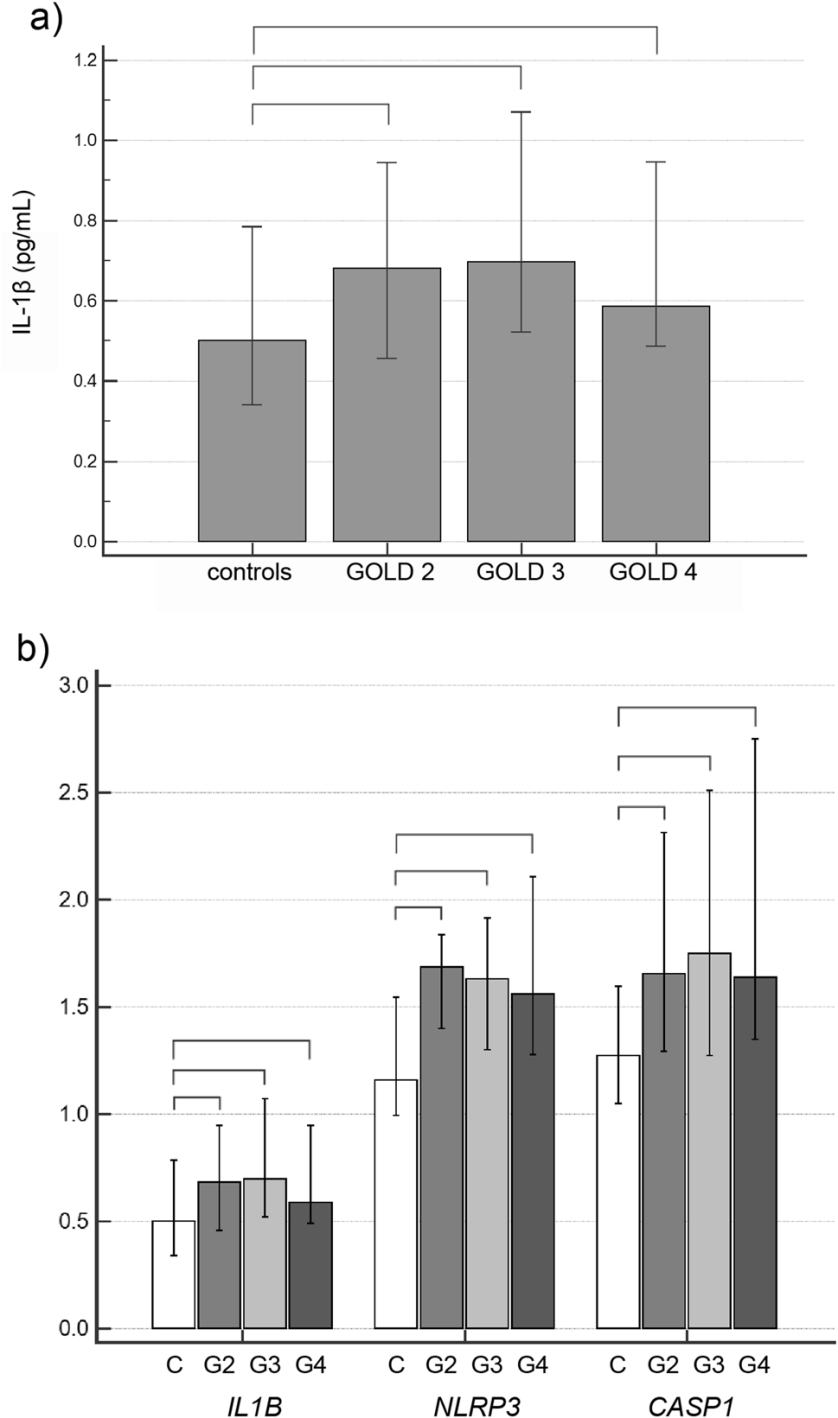
Plasma IL-1β concentration (**a**) and relative expression of *IL1B*, *NLRP3* and *CASP1* (**b**) in control and COPD subjects according to the severity of airflow obstruction. *IL-1β* interleukin-1beta, *IL1B* interleukin-1beta gene, *NLRP3* nucleotide-binding oligomerization domain-like receptor family pyrin domain-containing 3 gene, *CASP1* caspase-1 gene, *COPD* chronic obstructive pulmonary disease, *GOLD* Global Initiative for Chronic Obstructive Pulmonary Disease, *C* controls, *G2* GOLD 2, *G3* GOLD 3, *G4* GOLD 4. Data were tested by Kruskal–Wallis test, and results were presented as a median and IQR. If *P* < 0.05, post-hoc test was performed. Connectors are showing the differences with *P* < 0.05 between the groups after post-hoc analysis.

**Figure 3:**
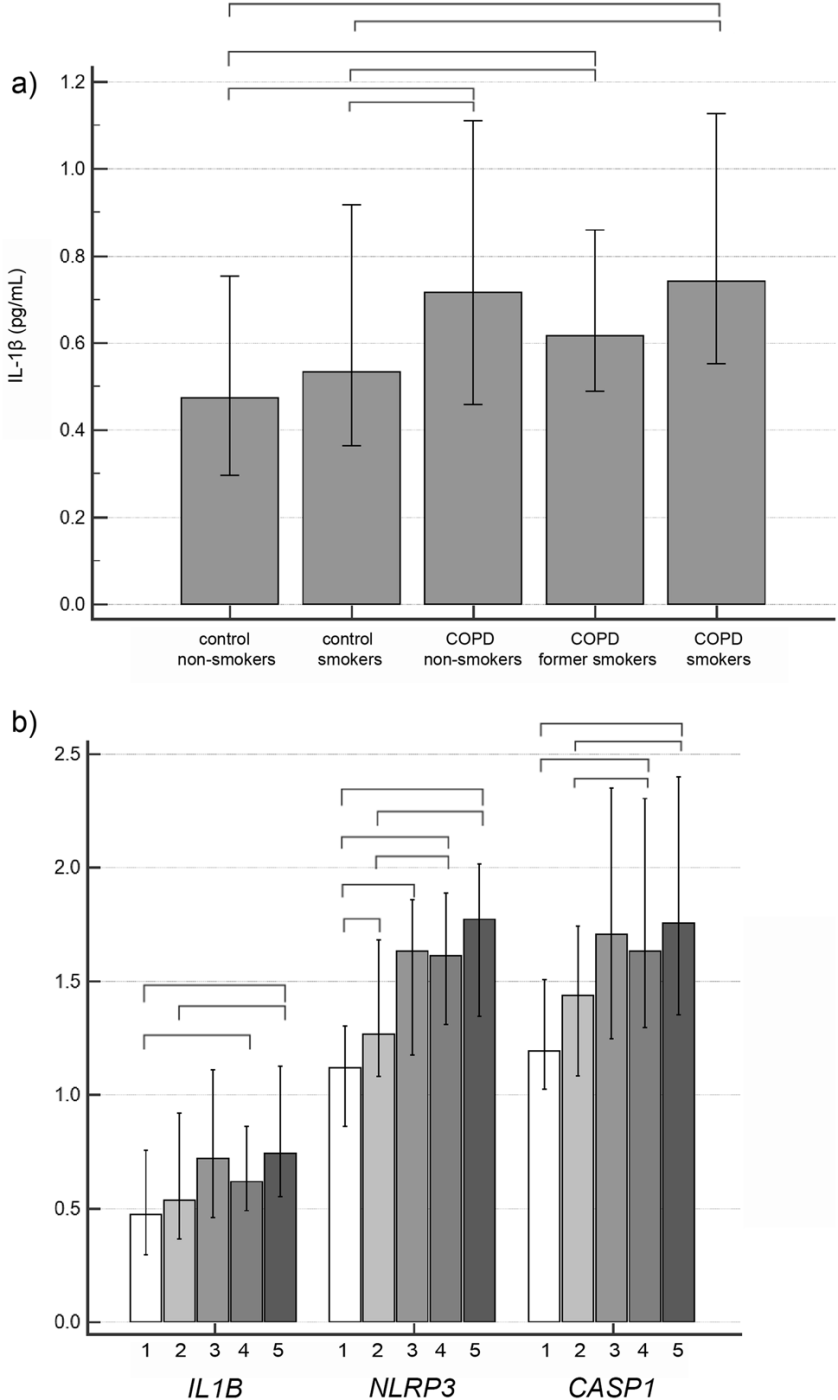
IL-1β plasma concentration (**a**) and relative expression of *IL1B*, *NLRP3* and *CASP1* (**b**) in healthy subjects and COPD patients when subdivided into the groups based on smoking status. *IL-1β* interleukin-1beta, *IL1B* interleukin-1beta gene, *NLRP3* nucleotide-binding oligomerization domain-like receptor family pyrin domain-containing 3 gene, *CASP1* caspase-1 gene, *COPD* chronic obstructive pulmonary disease; 1: control non-smokers; 2: control smokers; 3: COPD non-smokers; 4: COPD former smokers; 5: COPD smokers. Data were tested by Kruskal–Wallis test, and results were presented as a median and IQR. If *P* < 0.05, post-hoc test was performed. Connectors are showing the differences with *P* < 0.05 between the groups after post-hoc analysis.

Concentration of IL-1β was also significantly increased in plasma of patients with COPD (6.90 (0.61–23.91) pg/mL) when compared to healthy subjects (0.10 (0.10–0.23) pg/mL), *P* < 0.001. In addition, expression of *IL1B*, *NLRP3* and *CASP1* were increased in COPD patients in comparison to controls (Fig. 1). However, there was no significant difference in IL-1β concentration (Fig. 2a) or *IL1B*, *NLRP3* and *CASP1* expression (Fig. 2b) between COPD patients with different severity of the airway obstruction. In addition, the concentration of IL-1β and expressions of *IL1B*, *NLRP3* and *CASP1* were assessed based on smoking status in COPD patients and healthy volunteers (Fig. 3). Plasma concentration of IL-1β showed to be increased in all three COPD smoking groups in comparison to all healthy individuals (Fig. 3a). *NLRP3* gene expression showed to be increased in healthy smokers compared to healthy non-smokers. On the other hand, all three groups of COPD patients in terms of smoking status showed to have increased *NLRP3* mRNA levels in comparison to healthy non-smokers but only former and current COPD smokers had increased *NLRP3* expression compared to healthy smokers, while COPD non-smokers did not differ in *NLRP3* expression from healthy smokers. In addition, COPD non-smoking group was statistically similar to healthy subjects in terms of *IL1B* and *CASP1* mRNA levels. Expression of *IL1B* was increased in COPD former smokers in comparison to healthy non-smokers, and in COPD smokers in comparison to both healthy non-smokers and healthy smokers, while *CASP1* expression was increased in COPD former smokers and COPD smokers in comparison to both smoking groups of healthy volunteers (Fig. 3b).

### Predictive value of the selected parameters

Next, univariate logistic regression analysis was performed, and it showed that all examined parameters had statistically significant predictive potential for COPD according to the ORs and 95% CIs. Based on the results from univariate logistic regression analysis, multivariable logistic regression analysis was performed, and it showed that the combination of three parameters: IL-1β, FIB and WBC correctly classified 89% of cases. All results are summarized in Table 2.

**Table 2:** Univariate and multivariable logistic regression analyses of common inflammatory parameters, plasma IL-1β and gene expression of *IL1B*, *NLRP3* and *CASP1*.

Parameter	Univariate logistic regression analysis			Multivariable logistic regression analysis		
	OR	95% CI	*P* value	OR	95% CI	*P* value
CRP	1.24	1.09–1.41	**0.001**			
FIB	2.56	1.65–3.95	**< 0.001**	2.06	1.04–4.07	**0.040**
WBC	1.48	1.25–1.76	**< 0.001**	1.24	1.00–1.52	**0.050**
IL-1β	5.53	2.05–14.90	**< 0.001**	5.03	2.11–11.02	**< 0.001**
*IL1B*	2.25	1.25–4.05	**0.007**			
*NLRP3*	4.81	2.40–9.26	**< 0.001**			
*CASP1*	3.21	1.93–5.34	**< 0.001**			

Significant values are in bold.

*CRP* C-reactive protein, *FIB* fibrinogen, *WBC* white blood cells, *IL-1β* interleukin-1beta, *IL1B* interleukin-1beta gene, *NLRP3* nucleotide-binding oligomerization domain-like receptor family pyrin domain-containing 3 gene, *CASP1* caspase-1 gene, *OR* odds ratio, *CI* confidence interval.

## Discussion

Our study showed significantly increased plasma concentration of IL-1β as well as gene expression of *IL1B*, *NLRP3* and *CASP1* in COPD patients in stable phase compared to healthy controls, but there was no association with the severity of airflow obstruction or smoking status. In addition, IL-1β in the combination with WBC and FIB correctly classified 89% of all cases.

Several studies have investigated the systemic and local inflammatory state in COPD^19–22^, but the mechanisms of COPD pathogenesis are still poorly understood. Recent evidence suggests the involvement of the multiprotein complex NLRP3 inflammasome in the development of COPD^23^, but data regarding its activation are still scarce and contradictory. The present study showed that the plasma concentration of IL-1β is elevated in COPD patients compared to healthy controls, and the observed result is consistent with some previous studies that have investigated IL-1β in peripheral blood and in respiratory system samples^24,25^. However, in the study by Kleniewska et al. IL-1β was increased in the induced sputum of COPD patients, but there was no difference in its concentration in serum in comparison to healthy subjects^26^.

Faner et al. showed that *IL1B* and *NLRP3* mRNA was upregulated in lung tissue of stable COPD, but caspase-1 was mostly found in an inactive form, suggesting that the NLRP3 inflammasome was primed, but not activated^10^. On the contrary, in patients in acute exacerbation of COPD significantly increased gene expression of *IL1B*, *NLRP3* and *CASP1* as well as plasma concentration of IL-1β in peripheral blood was demonstrated^27^, whereas our study showed that similar processes also occurred in the stable phase of the disease. Given the lack of data on NLRP3 inflammasome in the systemic compartment of COPD patients, the present study could suggest that its activation may play an important role in the peripheral circulation as well as in the local respiratory compartment.

Regarding the severity of COPD, there was no association between *IL1B*, *NLRP3* and *CASP1* expression and the level of airflow obstruction in our study, and the same was observed for the plasma concentration of IL-1β. No association was found between plasma concentration of IL-1β and the severity of airflow obstruction in stable COPD in the study by Zou et al.^25^.

Cigarette smoke plays an important role in the development of inflammation^28^. To the best of our knowledge, there are no data on NLRP3 inflammasome activation in the peripheral circulation of COPD patients associated with patients’ smoking status. We found that expression of *IL1B*, *NLRP3* and *CASP1* as well as concentration of IL-1β amongst COPD patients were independent of their smoking history. However, results should be interpreted carefully and replicated analysis is suggested due to the small number of COPD non-smokers enrolled in the study.

Finally, we wanted to evaluate the diagnostic potential of the determined parameters. Based on the results of univariate logistic regression analysis, multivariable logistic regression analysis was performed, and it was found that the best model for COPD prediction is composed of IL-1β, FIB and WBC with correctly classified 89% of cases. However, a prospective and longitudinal study of IL-1β, WBC and FIB and of lung function should be performed to confirm potential clinical utility of these biomarkers in early identification of individuals at risk for developing COPD. This is important since COPD is a progressive lung condition with the significant impact on morbidity and mortality worldwide^29^.

The results of the study should be interpreted considering several limitations. First, a relatively small number of participants were included in the study, which affected the statistical power. Further studies should include more subjects, including those in the GOLD 1 stage of the disease as well as COPD non-smokers. However, COPD patients in GOLD 1 stage rarely contact physicians due to the mild symptoms, while most of the COPD patients are smokers (former or current), and, therefore, it is very difficult to recruit those specific subgroups. Currently, translating the signals into clinical practice and unravelling the complex interactions that lead to disease is challenging. Therefore, further studies are recommended with the aim to explore and explain COPD endotypes.

In conclusion, the results of our study suggest activation of NLRP3 inflammasome in the systemic compartment in patients with stable COPD, and also that the best model for COPD prediction might be the combination of three inflammatory parameters: IL-1β, FIB and WBC. Further investigations need to be designed to fully elucidate the comprehensive heterogeneity of COPD, which will enable to recognise novel diagnostic and/or therapeutic targets.

## Data Availability

The data presented in this study are available on request from the corresponding author.
